# Metagenomic analysis reveals the diversity of the vaginal virome and its association with vaginitis

**DOI:** 10.3389/fcimb.2025.1582553

**Published:** 2025-04-03

**Authors:** Xiang Lu, Qiang Lu, Rong Zhu, Mingzhong Sun, Hongmei Chen, Zhihu Ge, Yuchen Jiang, Zhipeng Wang, Lingzhi Zhang, Wen Zhang, Ziyuan Dai

**Affiliations:** ^1^ Department of Laboratory Medicine, School of Medicine, Jiangsu University, Zhenjiang, Jiangsu, China; ^2^ Department of Clinical Laboratory, Yancheng Third People’s Hospital, Affiliated Hospital 6 of Nantong University, The Affiliated Hospital of Jiangsu Medical College, Yancheng, Jiangsu, China

**Keywords:** metagenomics, anellovirus, papillomavirus, vaginitis, bacteriophage

## Abstract

**Introduction:**

The human vaginal virome is an essential yet understudied component of the vaginal microbiome. Its diversity and potential contributions to health and disease, particularly vaginitis, remain poorly understood.

**Methods:**

We conducted metagenomic sequencing on 24 pooled vaginal swab libraries collected from 267 women, including both healthy individuals and those diagnosed with vaginitis. Viral community composition, diversity indices (Shannon, Richness, and Pielou), and phylogenetic characteristics were analyzed. Virus–host associations were also investigated.

**Results:**

DNA viruses dominated the vaginal virome. *Anelloviridae* and *Papillomaviridae* were the most prevalent eukaryotic viruses, while *Siphoviridae* and *Microviridae* were the leading bacteriophages. Compared to healthy controls, the vaginitis group exhibited significantly reduced alpha diversity and greater beta diversity dispersion, indicating altered viral community structure. Anelloviruses, detected in both groups, showed extensive lineage diversity, frequent recombination, and pronounced phylogenetic divergence. HPV diversity and richness were significantly elevated in the vaginitis group, alongside an unbalanced distribution of viral lineages. Novel phage–bacterial associations were also identified, suggesting a potential role for bacteriophages in shaping the vaginal microbiome.

**Discussion:**

These findings provide new insights into the composition and structure of the vaginal virome and its potential association with vaginal dysbiosis. The distinct virome characteristics observed in women with vaginitis highlight the relevance of viral communities in reproductive health. Future studies incorporating individual-level sequencing and metatranscriptomics are warranted to explore intra-host viral dynamics, assess viral activity, and clarify the functional roles of vaginal viruses in host–microbiome interactions.

## Introduction

The vaginal microbiome, also known as the ‘vaginome’, is a critical component of the reproductive system microbiome and plays a significant role in women’s health and the progression of vaginitis and various diseases (Jakobsen et al., 2020). The vagina, as a direct interface with the external environment, offers warm and moist conditions that create an optimal habitat for microorganisms. Consequently, the viral communities inhabiting this microenvironment exhibit remarkable diversity and heterogeneity, shaped by factors such as individual physiology, lifestyle, sexual activity, and menstrual cycles (Wylie et al., 2018; Eskew et al., 2020; Huang et al., 2024). The anatomical and biochemical characteristics of the vagina confer natural defense mechanisms, including an acidic environment, epithelial barriers, immune responses, and microbial competition (Ravel et al., 2011; Chen et al., 2021). These factors collectively inhibit pathogen invasion and maintain microbial balance. However, disruptions to these defenses, caused by factors such as hormonal fluctuations, antibiotic use, or poor hygiene practices, can lead to an imbalance in the vaginal microbiome, thereby creating favorable conditions for pathogen colonization and the development of diseases such as vaginitis, which significantly affect reproductive health (Morsli et al., 2024).

Epidemiological studies have highlighted the important role of the cervical-vaginal microbiome in female reproductive health, particularly its association with adverse pregnancy outcomes such as preterm birth, premature rupture of membranes, intrauterine infections (Romero et al., 2007; Fettweis et al., 2019), as well as delivery methods, postpartum recovery, and newborn health (Kindinger et al., 2016; Vinturache et al., 2016), although the precise mechanisms remain unclear. Despite the limited understanding of the female vaginal microbiome, its relatively simple physiological structure and direct accessibility make the vagina an ideal system for investigating host-microbe interactions. Therefore, further investigation into the cervical-vaginal microbiome is expected to provide valuable insights into how microbial communities influence host health and may pave the way for strategies aimed at optimizing the vaginal microbiome to improve reproductive health in women globally.

Previous studies have shown that the vaginal virome encompasses diverse eukaryotic viruses, some of which are strongly associated with diseases such as cervical cancer (Chen et al., 2020; Bowden et al., 2023). Furthermore, recent research has revealed that the vagina harbors numerous previously unidentified viruses, which may be closely related to the onset and progression of diseases affecting the female reproductive system, representing a ‘viral dark matter’ (Liu et al., 2016; Jakobsen et al., 2020; Li et al., 2023). Compared to eukaryotic viruses, bacteriophages (phages) in the vaginal environment remain relatively understudied, with many phage genomes in women yet to be characterized. As bacterial-specific infective agents, phages are thought to play a critical role in shaping bacterial communities, maintaining ecological balance, and modulating the host immune system (Liu et al., 2016; Lawrence et al., 2019; Lu et al., 2023a).

Metagenomics is a powerful approach for studying the genetic material of all microorganisms in an environment, including both culturable and non-culturable microbes (Schloss and Handelsman, 2003). Traditional metagenomic research has primarily focused on bacteria and fungi, while viral sequences are often underrepresented due to their small genome size and low abundance in environmental microbial communities (Delwart, 2007). To bridge this gap, viral metagenomics has emerged as a specialized field for analyzing the diversity and functional potential of viral communities (Edwards and Rohwer, 2005; Delwart, 2007). It facilitates the identification of viruses in various environments, including those that infect humans and other vertebrates, as well as viruses present in diverse habitats such as soil and plant microbiomes, thereby enhancing our understanding of viral diversity, evolution, and ecological roles (Andersen et al., 2020; Lu et al., 2020; Shan et al., 2022; Wani et al., 2022).

Artificial intelligence (AI), particularly machine learning, has become an essential tool in metagenomics, playing a crucial role in identifying, classifying, and functionally annotating viral sequences (Hernandez Medina et al., 2022; Wani et al., 2022; Yan et al., 2024). AI-driven deep learning models and ensemble classifiers have been successfully employed for predicting virus-host interactions, reconstructing viral genomes, and improving classification accuracy (Yakimovich, 2021; Elste et al., 2024). Additionally, AI-driven algorithms have shown superior performance in detecting novel viral signatures in metagenomic datasets, substantially improving sensitivity and specificity over traditional bioinformatics pipelines (Rahimian and Panahi, 2024).

With the advancement of metagenomic technologies, research on the vaginal virome has advanced significantly, enabling systematic analysis of viral communities in specific microenvironments. This in-depth investigation not only helps establish baseline data for the female virome but also provides crucial insights into its role in women’s health, thereby deepening our understanding of viral ecology and its broader ecological implications (Schoenfeld et al., 2010).

In this study, we conducted a cross-sectional survey of the vaginal virome in 267 women, encompassing both healthy individuals and those diagnosed with vaginitis. Through metagenomic sequencing of vaginal swab samples, we aimed to characterize the overall composition and diversity of eukaryotic viruses and phages in the vaginal environment.

## Materials and methods

### Subjects and clinical data

To investigate the vaginal virome and its association with vaginitis, women who visited Affiliated Hospital 6 of Nantong University in January 2024 were enrolled in this study. The exclusion criteria were defined as ongoing pregnancy, immunosuppression due to medication, antibiotic use within the previous month, and a prior history of cervical treatment or surgery. Participants were classified into a healthy group and a vaginitis group based on colposcopy and microscopic examination of cervical secretions. The study included 137 patients with vaginitis and 130 healthy controls, with both groups further divided into 12 subgroups, each consisting of 10 to 12 individuals. All specimens were obtained from the Department of Clinical Laboratory, anonymized before analysis, and an exemption from informed consent was requested. The sample collection protocol was approved by the Ethics Committee of Affiliated Hospital 6 of Nantong University (Approval No. 2024-34).

### Sample collection, preparation, and sequencing

Vaginal swabs were obtained during consultations by the gynecologist through speculum examination. After inserting the speculum into the vaginal canal, swabs were used to collect samples from the anterior and posterior vaginal fornices as well as cervical secretions. Each vaginal swab was then placed in a sterile collection tube and immediately stored at 4°C. Before viral metagenomic analysis, the swab tips were immersed in 0.5 mL of Dulbecco’s phosphate-buffered saline (DPBS), vortexed vigorously for 5 minutes, and incubated for 30 minutes at 4°C. The supernatants were then collected after centrifugation at 15,000×g for 10 minutes and stored at -80°C until use.

Approximately 45 μL of supernatant from each vaginal swab specimen within the same subgroup was pooled together. Subsequently, the supernatant was filtered through a 0.45-μm filter (Millipore, Darmstadt, Germany) to remove eukaryotic, giant viruses and bacterial cell-sized particles. Filtrates were then digested by DNase and RNase at 37°C for 60 min. Total nucleic acids were then extracted using QIAamp MinElute Virus Spin Kit (Qiagen) according to the manufacturer’s protocol. Nucleic acid samples were dissolved in DEPC treated water and RNase inhibitors were added. The enriched viral nucleic acid preparations from the respective pools were individually subjected to reverse transcription reactions using reverse transcriptase (PureScript Enzyme, Vazyme) and 100 pmol of random hexamer primers, followed by a single round of DNA synthesis using Klenow fragment polymerase (New England BioLabs). A total of 24 libraries were constructed using TruePrep DNA Library Prep Kit (Vazyme) and subjected to sequencing on Illumina Novaseq 6000 platform.

### Metagenome assembly

To minimize host contamination, we downloaded the human reference genome (*Homo sapiens*, GCF_000001405.40) from NCBI and used Bowtie2 v2.4.5 (Langmead and Salzberg, 2012) for alignment and removal of potential host sequences from the 24 libraries. Primers and low-quality reads were trimmed using Trim Galore v0.6.5 (https://github.com/FelixKrueger/TrimGalore), with quality control performed using the specific options ‘*–phred33 –length 100 –stringency 3 –paired*’. Paired-end reads were assembled using MEGAHIT v1.2.9 (Li et al., 2016) with default parameters. To minimize false negatives during sequence assembly, the *De novo* assembler in Geneious Prime (https://www.geneious.com) was used to perform additional semi-automated assembly of unmapped reads and contigs shorter than 500 bp. After reassembly, contigs longer than 1500 bp were retained, while those with frame shifts were manually inspected and removed.

### Identification of viral genomes

We identified eukaryotic viruses and phage sequences through a series of steps. For eukaryotic viruses, all assembled contigs were aligned against the non-redundant protein (nr) database (downloaded on May 14, 2024) using the BLASTx program within DIAMOND v2.0.15 (Buchfink et al., 2021), filtering for significant contigs with an E-value cut-off of <10^–5^. Taxonomic identification of the significant contigs was conducted using the TaxonKit software (Shen and Ren, 2021). Contigs initially annotated as eukaryotic viruses were imported into Geneious Prime for manual assembly and examination, serving as the reference for mapping to the raw data using the Low Sensitivity/Fastest parameter. The resulting sequences were then screened for potential vector contamination using VecScreen (https://www.ncbi.nlm.nih.gov/tools/vecscreen) and clustered based on 95% nucleotide sequence identity and 90% coverage using MMseqs2 (*-k 0 -e 0.001 –min-seq-id 0.95 -c 0.9 –cluster-mode 0*) (Mirdita et al., 2019).

Phage identification was conducted on the remaining contigs after excluding those already identified as eukaryotic viruses. Contigs were validated using VirSorter2 (Guo et al., 2021) (*–min-length 3000*; *–min-score 0.5*) and then processed with CheckV (Nayfach et al., 2021a) to remove host sequences flanking prophages. Potential phage contigs were screened based on data from VirSorter2 and CheckV results, considering viral and host gene counts, VirSorter2 viral scores, and the presence of hallmark genes. These phage contigs were clustered with 95% average nucleotide identity (ANI) across 85% of the shortest contig, following MIUViG standards (Roux et al., 2019), using a custom script from the CheckV repository to define phage populations. The phage populations were further validated using VIBRANT (Kieft et al., 2020), and only the results consistent between VIBRANT and VirSorter2 were retained. The phage populations were subsequently aligned with the nr database for taxonomic classification. To maximize the acquisition of taxonomic information on unannotated phages, we used BLASTn (v2.15.0) (Camacho et al., 2009) to search all sequences against an additional set of public viral databases, including the Gut Virome Database (GVD) (Gregory et al., 2020), Gut Phage Database (GPD) (Camarillo-Guerrero et al., 2021), Metagenomic Gut Virus Catalog (MGV) (Nayfach et al., 2021b), and the Chinese Human Gut Virome Catalogue (CHGV) (Chen et al., 2024). We annotated sequences that had both alignment identity and coverage greater than 90% to the subject sequences. A phage sequence was considered novel if it had <95% ANI relative to other viral sequences or if it did not align with any known sequences. Phage populations were categorized into genus-level viral taxa through a gene-sharing network analysis using vContact2 (Bin Jang et al., 2019), with NCBI RefSeq Viral (release 211) serving as the reference genomes. The clustered contig networks were displayed using Cytoscape v3.10.3 (Shannon et al., 2003). The overall pipeline of virome analysis is illustrated in **Supplementary Figure 1**.

### Viral genome annotation

Geneious Prime was employed to predict putative open reading frames (ORFs) using parameters of a minimum size of 100 bp and an ATG start codon. These ORFs were subsequently validated by comparing them against similar viruses in the GenBank database. ORF annotations were assigned by referring to the CDD v3.21 database within the Conserved Domain Database (CDD) (Wang et al., 2023), which incorporates NCBI-curated domains alongside data from Pfam, SMART, COG, PRK, and TIGRFAM. GraPhlAn was used to visualize the viral taxonomy diagram at taxonomic levels ranging from realm to genus, following the methodology provided in the GraPhlAn tutorial available at https://huttenhower.sph.harvard.edu/GraPhlAn.

### Phylogenetic analysis

To infer phylogenetic relationships, nucleotide sequences of reference strains belonging to different groups of viruses were downloaded from the NCBI GenBank database. Related nucleotide sequences were aligned using an alignment program implemented in Geneious Prime. The maximum-likelihood phylogenetic trees were constructed from the alignment using IQ-TREE (Nguyen et al., 2015). The best-fitting model was identified by ModelFinder (Kalyaanamoorthy et al., 2017).

### Recombination analyses

A single representative of the three anellovirus genera was chosen for a closer analysis, giving clusters with 12 *Alphatorquevirus*, 10 *Betatorquevirus*, and 11 *Gammatorquevirus* sequences. These three clusters, where all members were at least 60% identical to another member at the nucleotide level. Next, sequences within each cluster were realigned with MAFFT to improve the alignments. Then, each alignment was split into 500-nucleotide fragments, and phylogenies were inferred from each fragment using IQ-TREE and midpoint-rooted. Phylogenies derived from neighboring fragments were then displayed in a tangled chain where each taxon is tracked through successive trees. Robinson-Foulds distances between neighboring trees were computed with the ETE 3 toolkit (Huerta-Cepas et al., 2016).

The same cluster alignments, undivided, were used to infer single trees with IQ-TREE. Each tree and alignment were then used to reconstruct the mutations that occurred using the tree with ClonalFrameML (Didelot and Wilson, 2015)with kappa set to 2.0. For every mutation that was reconstructed to occur only once in the tree, the branch where the mutation occurred was marked with ticks. Mutations inferred to occur more than once were indicated by a line connecting them to their identical counterparts elsewhere in the tree (i.e., reversions were considered separately).

Finally, we inferred the decay of LD by using the χ^2df^ statistic (Hedrick and Thomson, 1986), which behaves identically to the more common r^2^ statistic for biallelic loci. To this end, we used the genus-wide alignments with 161 *Alpha*-, 99 *Beta*-, and 77 *Gammatorquevirus* sequences. Alignment columns with fewer than 10% valid sites (A, C, T, or G) were ignored, as were sites where the minority variant was at lower than 5% frequency. LD measured between pairs of variable sites was then plotted against the distance between sites, with mean LD calculated in 100-nucleotide windows.

### Pairwise sequence identity analysis

Pairwise identity comparisons at the nucleotide level of all anellovirus lineages were computed with the Sequence Demarcation Tool (SDT) (Muhire et al., 2014). Each set of sequences was aligned using SDT with the MAFFT option. Pairwise amino acid identity (AAI) comparisons between anellovirus lineages were computed with the CompareM toolkit (https://github.com/dparks1134/CompareM). All 125 lineages derived from the anellovirus cohort were first split into individual FASTA files with the *seqkit* sp*lit* command with the *-i* parameter to split by sequence identifier. The directory containing these FASTA files was used as input to CompareM’s *aai_wf* command to compute the mean AAI values between each lineage.

## Results

### Overview of the vaginal virome

**Figure 1:**
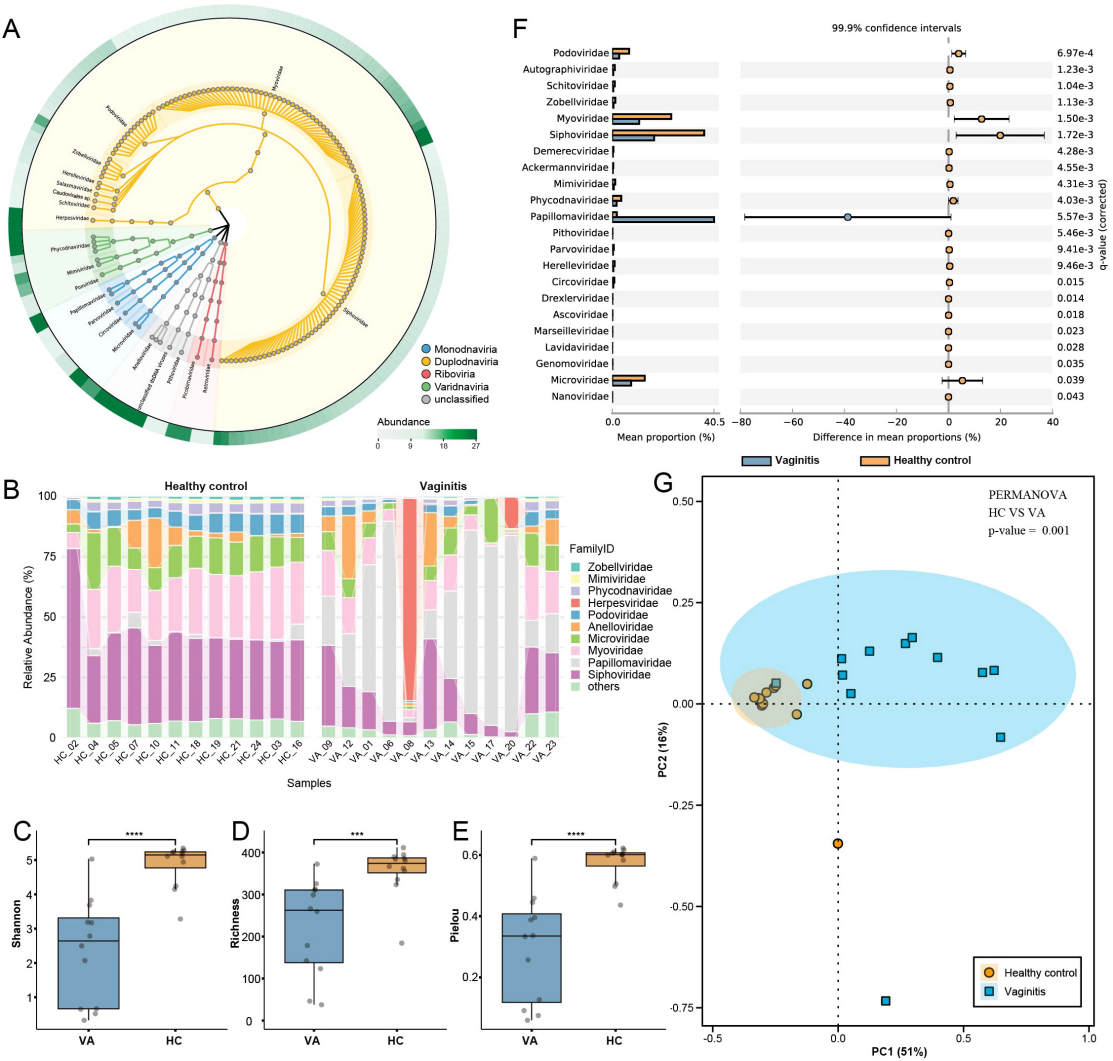
Overview of viral community structure and diversity in healthy controls and vaginitis groups. **(A)** Viral taxonomy tree visualized using GraPhlAn, constructed from the 150 most abundant viral genera across all libraries. Background colors indicate different viral realms, highlighting taxonomic diversity. **(B)** Family-level relative abundance of the vaginal virome displayed as a percentage stacked bar chart, comparing healthy control and vaginitis groups. **(C–E)** Alpha diversity indices (Shannon, Richness, and Pielou) comparing the healthy control and vaginitis groups. Statistical significance was determined using the Wilcoxon rank-sum test. Exact *p*-values: Shannon index (*p* = 0.00014), Richness (*p* = 0.00066), Pielou index (*p* = 0.00014). **(F)** STAMP analysis showing differences in the relative abundance of viral families between healthy control and vaginitis groups. The mean proportions and 99.9% confidence intervals are displayed. **(G)** Beta diversity analysis based on Bray-Curtis distances, visualized through PCoA. The percentages of variance explained by PC1 and PC2 are indicated on the respective axes. Ellipses represent the 95% confidence intervals for each group. Statistically significant separation between the two groups was confirmed using PERMANOva (*p* < 0.01). Statistical significance thresholds: *p < 0.05, **p < 0.01, ***p < 0.001,****p < 0.0001.

Genome sequencing was performed on 24 vaginal swab libraries using the Illumina NovaSeq 6000 platform, generating a total of 12,124,387 viral reads after trimming, quality control, and the exclusion of human and bacterial sequences. The 150 most abundant viruses at the genus level were selected to construct a viral taxonomy diagram using GraPhlAn, as illustrated in **Figure 1A**. Among these, 130 genera were assigned to the realm *Duplodnaviria*, with the majority belonging to the families *Myoviridae* and *Siphoviridae*. Notably, high abundances were observed in *Microviridae* and *Papillomaviridae* within the realm *Monodnaviria*, *Picobirnaviridae* in *Riboviria*, and *Anelloviridae*, which has not yet been classified into any realm. Viruses were quantified and normalized to investigate compositional differences between vaginal samples from the healthy control and vaginitis groups at the family level. In the healthy control group, *Siphoviridae* (36.58%), *Myoviridae* (23.53%), and *Microviridae* (12.88%) were the most predominant families. Conversely, in the vaginitis group, *Papillomaviridae* (40.53%) exhibited the highest relative abundance, followed by *Siphoviridae* (16.73%) and *Myoviridae* (10.75%) (**Figure 1B**). Statistical Analysis of Metagenomic Profiles (STAMP) (Parks et al., 2014) was used for statistical hypothesis testing and exploratory data visualization. In this study, STAMP identified significant differences in family-level relative abundance within the vaginal virome between the vaginitis and healthy control groups. Specifically, 22 viral families were differentially abundant between the two groups, as illustrated in **Figure 1F**. Alpha diversity was evaluated using Shannon, Richness, and Pielou indices. The analysis revealed that all three indices were significantly higher in the healthy control group compared to the vaginitis group (**Figures 1C–E**). Statistical significance was confirmed using the Wilcoxon rank-sum test (*p* < 0.05). Principal coordinate analysis (PCoA) based on Bray-Curtis distances was performed to evaluate beta diversity. The results indicated that the vaginitis group exhibited substantially greater sample dispersion compared to the healthy control group, reflecting significant differences in beta diversity between the two groups (**Figure 1G**). These differences were further validated through PERMANOVA, which demonstrated a statistically significant separation between the vaginitis and healthy control groups (*p* < 0.01).

### Diversity of *Anelloviridae* in the human vagina

**Figure 2:**
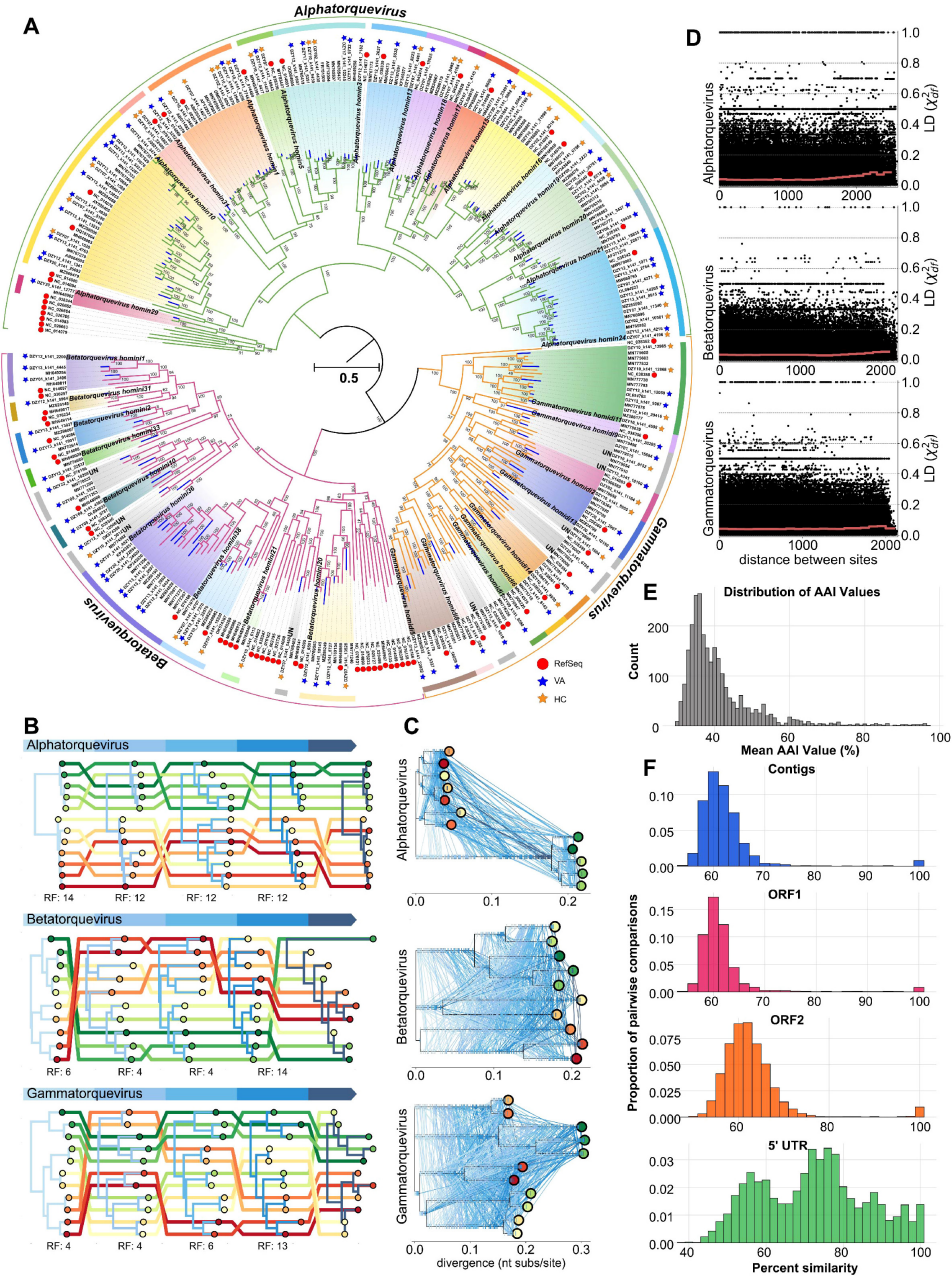
Diversity and recombination in anelloviruses **(A)** Maximum-likelihood phylogeny of anellovirus ORF1 nucleotide sequences (n = 337). The phylogenetic tree highlights the diversity of anellovirus lineages with distinct clades representing different genera. **(B)** Tangled chain of midpoint-rooted phylogenies inferred from 500-nucleotide fragments of the anellovirus ORF1. Tree colors denote the index positions of fragments within the alignment, and tips of the same branch are connected by lines of distinct colors. Labels below the trees indicate the Robinson-Foulds (RF) distances between neighboring trees. **(C)** Ancestral sequence reconstruction tree with lines connecting identical mutations on different branches. Proportions along branch lengths represent the relative positions of mutations in the genome, while ticks on branches indicate unique mutations that are exclusive to the respective branches. **(D)** Decay of linkage disequilibrium (LD) across genomic distances within anellovirus genera. LD values (χ^2df^) were plotted against distances between polymorphic sites, showing effective free recombination across adjacent loci. **(E)** Pairwise amino acid identity (AAI) comparisons between anellovirus lineages. ORFs from each lineage were compared to quantify interhost lineage similarity. **(F)** Distribution of pairwise identities across the dataset, categorized into full contigs, ORF1 protein, ORF2 protein, and 5’ UTR regions, highlighting the variability in similarity among these features.

Anelloviruses were detected in most healthy individuals and vaginitis patients, with co-infections of multiple unique lineages within the same sample, as well as infections of the same lineage across multiple individuals, were frequently observed (Rani et al., 2016; Moustafa et al., 2017; Bal et al., 2018). The *Anelloviridae* family consists of non-enveloped viruses with circular, negative-sense, single-stranded DNA (ssDNA) genomes ranging from 1,600 bp to 3,900 bp in length (Varsani et al., 2021). In this study, a total of 125 anelloviruses were identified, with genome sizes ranging from 2,090 bp to 3,737 bp. Each genome contained a large ORF1 encoding the nucleocapsid protein, which is rich in arginine (Arg) at the N-terminus. The analysis of alpha diversity revealed no significant differences between the healthy control and vaginitis groups (**Supplementary Figure 2**). The *Anelloviridae* family found in the human vagina predominantly comprised three genera: *Alphatorquevirus* (n = 70), *Betatorquevirus* (n = 30), and *Gammatorquevirus* (n = 25), which exhibited an average pairwise AAI of approximately 41% (**Figure 2E**). We found that the 5’ UTR exhibited the highest average pairwise similarity at 71%, compared to approximately 62% observed in full contigs, ORF1 protein sequences, and ORF2 protein sequences (**Figure 2F**).

As illustrated in **Figure 2A**, the phylogenetic tree based on the ORF1 gene revealed that a total of 109 anelloviruses identified in this study clustered with members of 33 species within the genera *Alphatorquevirus*, *Betatorquevirus*, and *Gammatorquevirus*. In contrast, the remaining 16 anelloviruses formed distinct clades that did not align with any previously recognized species. Notably, the phylogenetic branch length for newly added sequences was more pronounced in the *Betatorquevirus* (0.100 substitutions per nucleotide site) and *Gammatorquevirus* (0.129) genera compared to *Alphatorquevirus* (0.043), as indicated by the blue-colored branches. These results suggest that the diversity of *Alphatorquevirus* has been largely explored, whereas each newly added *Betatorquevirus* and *Gammatorquevirus* sequence contributes substantial previously uncharted diversity to the phylogenetic tree. Phylogenetic analyses of 500-nucleotide segments of aligned ORF1 sequences revealed inconsistent topologies, suggesting possible recombination events (**Figure 2B**). Frequent homoplasy was identified across three reconstructed trees through ancestral sequence reconstruction, as shown in **Figure 2C**. Linkage disequilibrium (LD) values were calculated and correlated with genomic distances within each genus to quantify recombination in the ORF1 sequence. The analysis showed that LD values between polymorphic sites, particularly those at adjacent positions, averaged near zero, indicating efficient free recombination (**Figure 2D**).

### Vaginal papillomavirus diversity analysis

**Figure 3:**
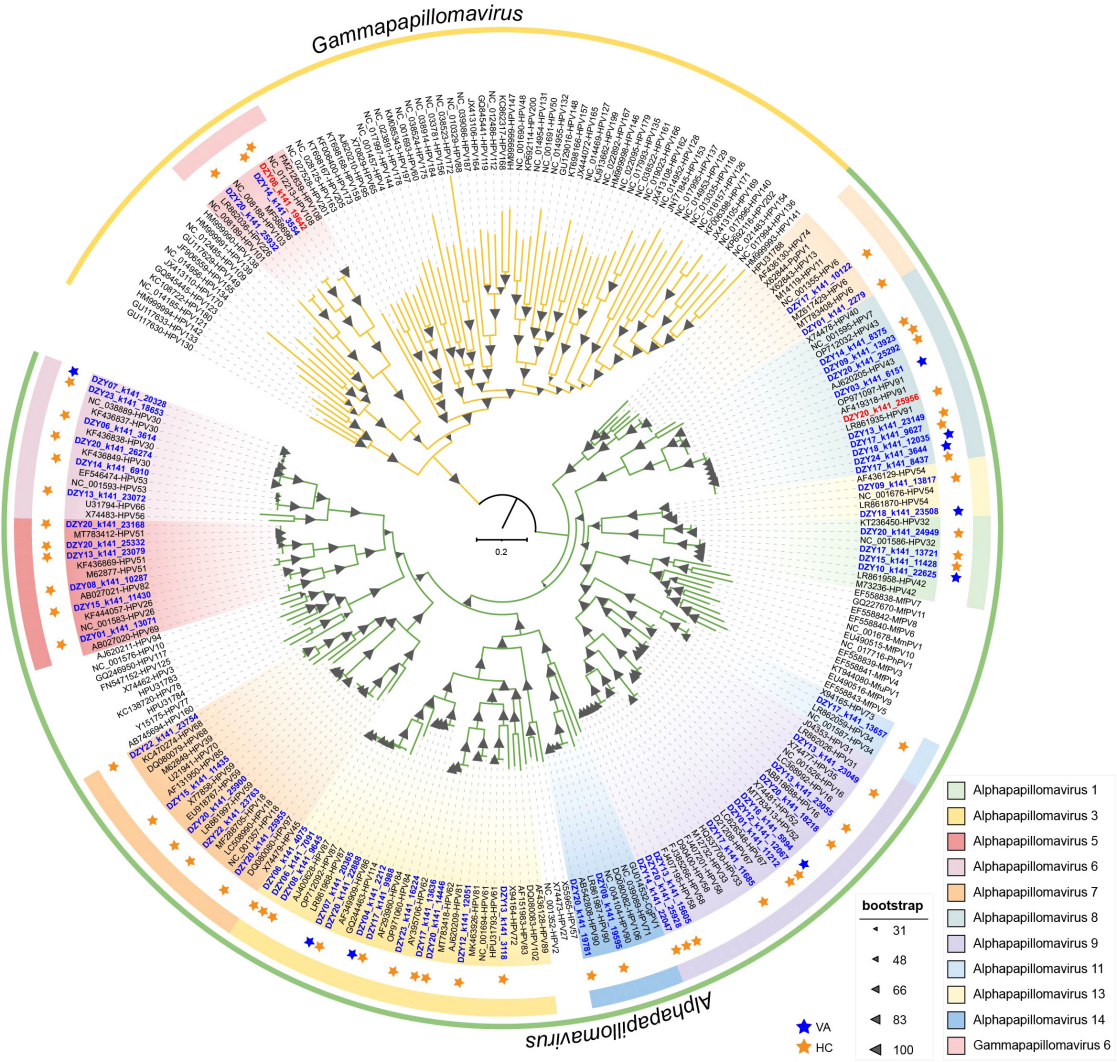
Maximum-likelihood phylogeny of papillomavirus L1 nucleotide sequences (n = 252). The phylogenetic tree highlights the diversity of papillomavirus lineages with distinct clades representing different species.

Members of the *Papillomaviridae* family are non-enveloped viruses with small, circular, double-stranded DNA (dsDNA) genomes ranging from 5,700 bp to 8,600 bp in length (Lu et al., 2023b). A total of 63 human papillomavirus (HPV) genomes were obtained from female vaginal swab samples, with genome sizes ranging from 5,974 bp to 7,857 bp. Among these, 48 were identified as complete genomes, containing a complete L1 gene. Based on genomic classification, 60 isolates were assigned to the *Alphapapillomavirus* genus, while three were classified as *Gammapapillomavirus*, spanning across 12 species. The L1 ORF sequence was used to categorize these genomes into 31 distinct HPV types (Chen et al., 2018). Phylogenetic analysis based on the L1 gene revealed that papillomavirus sequences obtained in this study clustered within known HPV species (**Figure 3**). The identified sequences exhibited distinct phylogenetic clustering, with multiple newly identified strains forming unique branches. Bootstrap support values indicated robust phylogenetic placement, further confirming the classification of these genomes. The distribution of sequences between the healthy control and vaginitis groups revealed that viral lineages from the vaginitis group exhibited greater diversity compared to the healthy control group.

Alpha diversity analyses revealed significant differences in the papillomavirus community composition between the healthy control and vaginitis groups (**Supplementary Figure 3**). Pielou’s evenness index was significantly lower in the vaginitis group compared to the healthy control group, indicating a more uneven distribution of viral lineages in the vaginitis group. The Shannon diversity index, which accounts for both richness and evenness, showed no significant difference between the two groups. However, species richness was significantly higher in the vaginitis group than in the healthy control group, suggesting that individuals with vaginitis harbor a greater number of papillomavirus lineages. These findings indicate that while the overall diversity (Shannon index) remained comparable, the vaginitis group harbored a more diverse set of viral lineages with a less even distribution.

These findings highlight the diversity of vaginal papillomaviruses and suggest that individuals with vaginitis may harbor a broader range of HPV lineages, potentially influencing viral ecology within the vaginal microbiome.

### Diversity, classification, and host associations of vaginal phages

**Figure 4:**
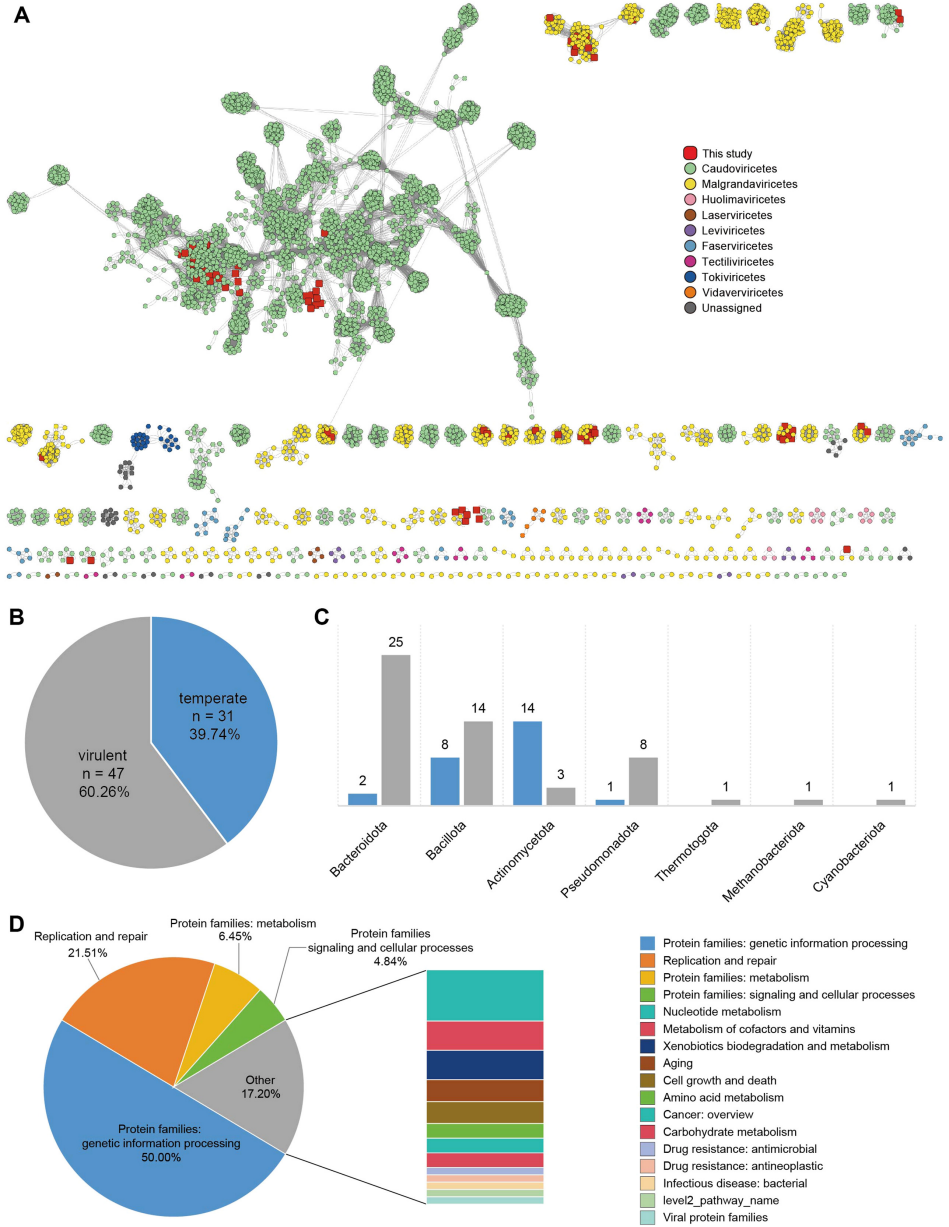
Classification and host predictions of vaginal phages **(A)** Viral clustering network analysis. A gene-sharing network of identified phage populations was constructed using vContact2, with taxonomic assignments based on NCBI RefSeq Viral (release 211). Each node represents a phage genome, and edges indicate shared gene content between phages. **(B)** Lifestyle classification of phages. Phages were classified as virulent or temperate based on PhaTYP and CHERRY predictions. **(C)** Predicted host distribution of phages. **(D)** Functional classification of phage-associated proteins based on the eggNOG database.

A total of 78 phage sequences were identified and subsequently clustered into 5,629 phage populations along with known viruses from other databases. Among them, 12 phages were classified as singletons or outliers, while the remaining 66 phage populations exhibited overlapping cluster assignments, indicating their association with multiple viral clusters (**Figure 4A**). To further classify these phage populations, protein clustering was performed using vContact2, which assigned the 78 phages to 54 viral clusters. Of these, 40 clusters were classified as *Microviridae* within *Malgrandaviricetes*, while 38 clusters were assigned to *Caudoviricetes*. Notably, these phage populations were not assigned to any known genera, suggesting the presence of novel viral lineages. Phage host linkages and lifestyles were predicted using PhaTYP and CHERRY suites within PhaBOX under default parameters (Shang et al., 2023). The identified novel phages were associated with seven bacterial phyla, predominantly *Bacteroidota* (n=27), *Bacillota* (n=22), and *Actinomycetota* (n=17) (**Figure 4C**). Phages infecting *Bacteroidota* and *Bacillota* were primarily virulent, whereas those infecting *Actinomycetota* were predominantly temperate (**Figure 4B**). These findings highlight the diverse ecological roles of vaginal phages, revealing both novel viral lineages and distinct host associations within the vaginal virome. Based on functional annotation using the KEGG search program in eggNOG-mapper v2 (Cantalapiedra et al., 2021), the majority of detected genes in phage sequences were associated with genetic information processing (50.00%) and replication and repair (21.51%), highlighting their roles in maintaining and regulating genetic material. Additionally, metabolic pathways (6.45%), including those related to nucleotide metabolism, amino acid metabolism, and cofactor metabolism, were identified. A smaller proportion of genes were linked to signaling and cellular processes (4.84%), while 17.20% of the annotated genes belonged to miscellaneous functional categories (**Figure 4D**; **Supplementary Table 5**). These findings suggest that phage-encoded genes play a crucial role in genetic regulation and host interactions, with a subset contributing to metabolic functions.

Additionally, we found that the diversity of these novel identified phages was slightly higher in the vaginitis group compared to the healthy group (**Supplementary Figure 4**), suggesting that these phages were more diverse and evenly distributed in the inflammation-associated samples. However, this difference was not statistically significant, which may indicate that their overall abundance was relatively low and thus had no substantial impact on the viral community structure. Furthermore, under inflammatory conditions, a greater variety of low-abundance phages may emerge rather than a community dominated by a single species.

## Discussion

Although this study employed a method capable of detecting both DNA and RNA viruses to analyze the vaginal virome, the results indicate that the predominant viral components in the vagina are DNA viruses, consistent with previous studies (Rahimian and Panahi, 2024). Among these, *Anelloviridae* and *Papillomaviridae* were the most prevalent eukaryotic viruses, while *Siphoviridae* and *Microviridae* were the most abundant phages. In women with vaginitis, the composition of the vaginal virome exhibited significant alterations. Compared to the healthy control group, the alpha diversity indices (Shannon, Richness, and Pielou indices) were significantly lower in the vaginitis group, whereas beta diversity analysis revealed a higher degree of dispersion in the vaginitis group, reflecting significant differences in virome composition between the two groups. These findings are consistent with the limited research investigating the relationship between the vaginal virome and female reproductive health. For instance, higher eukaryotic viral diversity has been linked to preterm birth and reproductive outcomes in asymptomatic women (Wylie et al., 2018; Eskew et al., 2020). Similarly, in patients with bacterial vaginosis, eukaryotic viral abundance was elevated, while changes in phage composition were associated with bacterial community characteristics and bacterial vaginosis status (Jakobsen et al., 2020).

Anelloviruses are highly diverse and have been detected in both healthy individuals and those with various conditions, raising questions about their ecological roles and potential pathogenicity. Their widespread presence and frequent co-infections suggest an adaptive capacity to diverse host environments. As viral loads are often higher in individuals with immunodeficiencies (Sajiki et al., 2023; Boukadida et al., 2024; Esser et al., 2024), anelloviruses are likely under immunological control. This study contributes to the expanding taxonomy of the *Anelloviridae* family, identifying unclassified lineages that may represent novel species. Given the International Committee on Taxonomy of Viruses (ICTV) criterion of <69% pairwise ORF1 similarity to define distinct species (Varsani et al., 2021), the uncharacterized sequences from the vaginal virome highlight the need for continued refinement of anellovirus taxonomy, particularly as more diverse genomes are deposited through metagenomic studies. Recombination emerged as a key driver of anellovirus diversity, evident from inconsistent phylogenetic topologies and near-zero linkage disequilibrium within ORF1 sequences. This underscores the evolutionary plasticity of anelloviruses, enabling them to adapt and persist in complex host ecosystems like the vaginal microbiome. These findings emphasize the importance of further research into the functional and ecological significance of anelloviruses within the human vaginal virome. Understanding their interactions with host immunity and co-infecting microbes will be critical for elucidating their roles in health and disease.

This study provides a comprehensive analysis of vaginal HPVs, revealing diverse viral lineages with distinct community structures between healthy individuals and those with vaginitis. The significantly higher species richness observed in the vaginitis group suggests a broader range of HPV infections in individuals with vaginal dysbiosis. However, the lower Pielou’s evenness index indicates an unbalanced distribution of these viral lineages. This supports previous findings that dysbiotic vaginal environments often harbor increased viral diversity, potentially influenced by altered host immunity and microbial interactions (Mitra et al., 2015, 2016). Despite no novel lineages being identified, phylogenetic analysis confirmed the presence of a wide range of HPV types, predominantly within *Alphapapillomavirus* and *Gammapapillomavirus*. The functional implications of this diversity remain unclear, particularly regarding its role in vaginal health and disease progression. While some HPV types are well-established in cervical pathology, their impact on vaginal microbiome stability requires further investigation (Brotman et al., 2014). A limitation of this study is the lack of functional assessment, such as viral transcriptional activity or host immune response profiling. Future studies should explore how HPV diversity contributes to vaginal dysbiosis and whether it serves as a biomarker for disease risk. Expanding metagenomic analyses to include functional annotation will be crucial in understanding HPV-host interactions (Di Paola et al., 2017).

Phage-encoded functional genes influence host metabolism and environmental adaptation while also playing key roles in ecosystem dynamics and microbial community interactions (Nick et al., 2022; Feng et al., 2024). Although not essential for phage replication, these genes can regulate or enhance host metabolic activities, creating a more favorable environment for phage proliferation and dissemination. The differences in phage diversity between the vaginitis and healthy groups, while not statistically significant, provide intriguing clues about the role of phages in vaginal microbial dynamics. The slightly higher diversity of newly identified phages in the vaginitis group may reflect an increased turnover of viral populations due to microbial shifts associated with inflammation. Inflammatory conditions could alter the phage-host interactions, either by driving the selection of certain bacterial hosts or by promoting the activation of temperate phages through stress-induced prophage induction. Furthermore, our study followed the viral taxonomy established by the ICTV prior to its 2022 revision, which classified tailed phages, including those in the families *Siphoviridae*, *Myoviridae*, and *Podoviridae*, based on tail morphology (Turner et al., 2023). However, the ICTV’s update reclassified these groups within the newly designated class *Caudoviricetes*. To ensure comparability with previous research on host associations and ecological distribution, this study retains the original classification. It is important to note that while this taxonomic revision reflects a re-evaluation of genomic relationships, the functional characteristics of viruses, such as host specificity and lytic/lysogenic behavior, as well as tail morphology, remain valuable phenotypic markers. Therefore, the core findings of this study are not substantially affected by the updated taxonomic framework.

Although sample pooling is a well-established strategy in metagenomic studies that enhances sequencing efficiency, maximizes sequencing depth, and increases data yield, it inherently limits the ability to perform individual-level correlation analyses with clinical metadata, such as symptom severity, hormonal status, and sexual activity. This trade-off is particularly relevant in virome studies, as host-specific factors can significantly influence virome composition. However, the primary objective of this study was to provide a comprehensive characterization of the vaginal virome’s composition and diversity rather than to investigate inter-individual differences. While our approach facilitated the identification of diverse viral taxa and offered a broad overview of the vaginal virome, future studies should incorporate individual-level sequencing to capture intra-host viral variability and characterize personalized virome dynamics. Additionally, integrating single-sample sequencing with high-resolution bioinformatics and AI-driven approaches could further enhance our understanding of viral diversity and its potential associations with host health (Yakimovich, 2021; Elste et al., 2024). Another limitation of this study is the lack of metatranscriptomic and proteomic validation, which prevents us from determining whether the detected viruses are actively replicating or have been integrated into the microbiome. While metagenomic sequencing provides a comprehensive snapshot of viral genetic material, it does not distinguish between active and latent viruses. Future studies should incorporate metatranscriptomic and proteomic analyses to confirm viral activity, explore gene expression patterns, and assess the functional contributions of the virome. These approaches will provide deeper insights into the ecological roles of vaginal viruses and their potential impact on microbial community dynamics and host health.

## Conclusion

This study provides a comprehensive metagenomic analysis of the vaginal virome, revealing distinct viral community compositions between healthy and vaginitis groups. DNA viruses, particularly *Anelloviridae* and *Papillomaviridae*, dominated the eukaryotic virome, while *Siphoviridae* and *Microviridae* were the most abundant phages. The vaginitis group exhibited lower alpha diversity and greater beta diversity dispersion, indicating virome shifts associated with dysbiosis. Novel phage-bacterial interactions suggest a potential role of phages in shaping the vaginal microbiome. Future studies should incorporate individual-level sequencing and functional analyses to assess viral replication, host interactions, and its impact on reproductive health. These approaches will further advance our understanding of the ecological and clinical significance of the vaginal virome.

## Data Availability

The datasets presented in this study are publicly available in the NCBI Sequence Read Archive (SRA) under the BioProject accession number PRJNA1170175. These data can be accessed through the NCBI SRA database.
