# Supplementary material for: Metagenomic analysis reveals the diversity of the vaginal virome and its association with vaginitis
Source: Front Cell Infect Microbiol. 2025 Apr 3;15:1582553. doi: 10.3389/fcimb.2025.1582553 (PMC12003417; doi:10.3389/fcimb.2025.1582553)

Supplementary Figure 1. Pipeline for virome analysis. The workflow illustrates the key steps in metagenomic sequencing, quality control, viral genome assembly, classification, and downstream analyses.

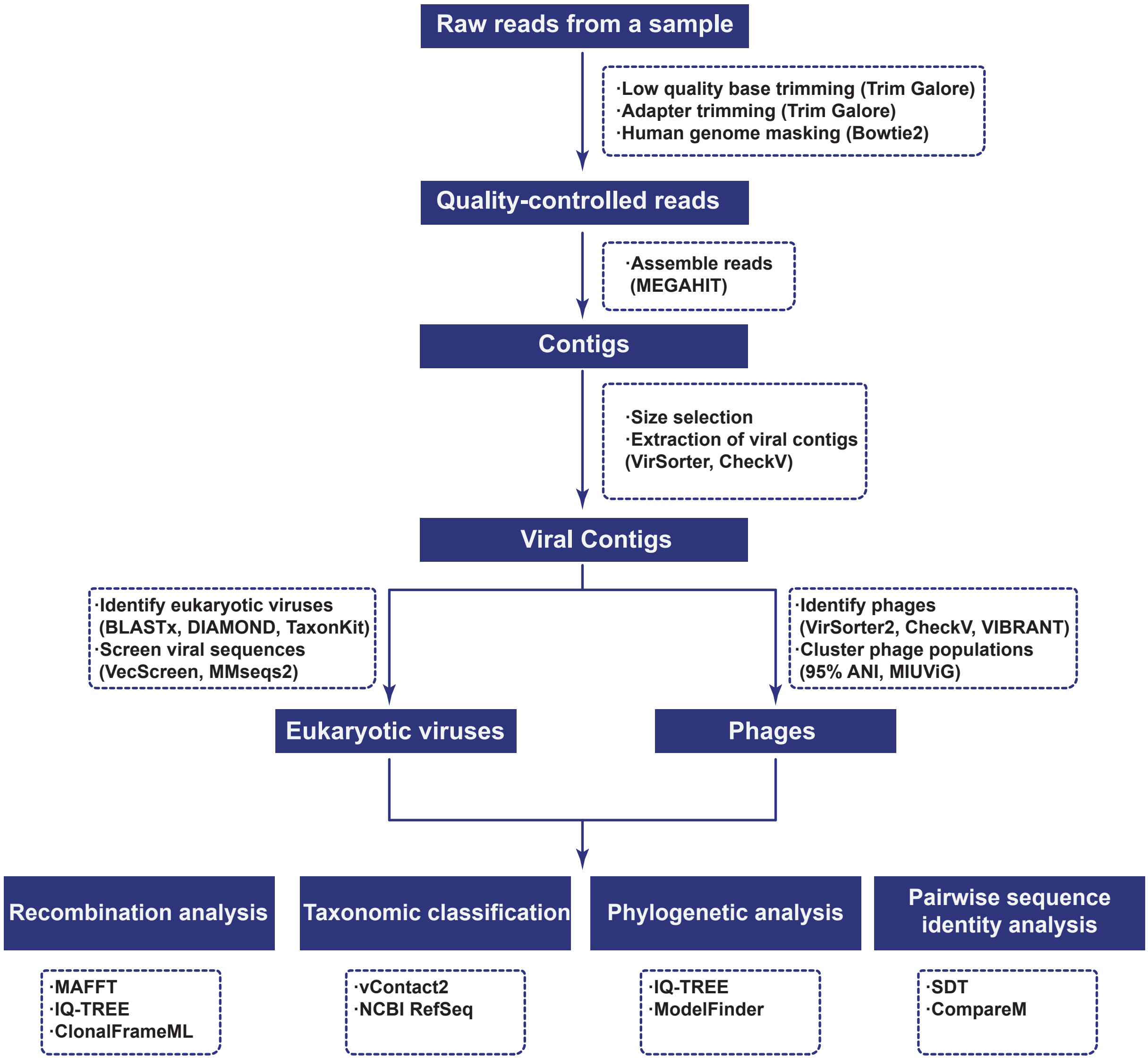

Supplementary Figure 2. Alpha diversity indices (Pielou, Shannon, and Richness) of all 125 anellovirus-derived lineages, comparing the healthy control and vaginitis groups. Statistical significance was determined using the Wilcoxon rank-sum test. Exact *p*-values: Shannon index ($p = 0.95$), Richness ($p = 0.27$), Pielou index ($p = 0.76$). Statistical significance thresholds: *$p < 0.05$, **$p < 0.01$, ***$p < 0.001$.

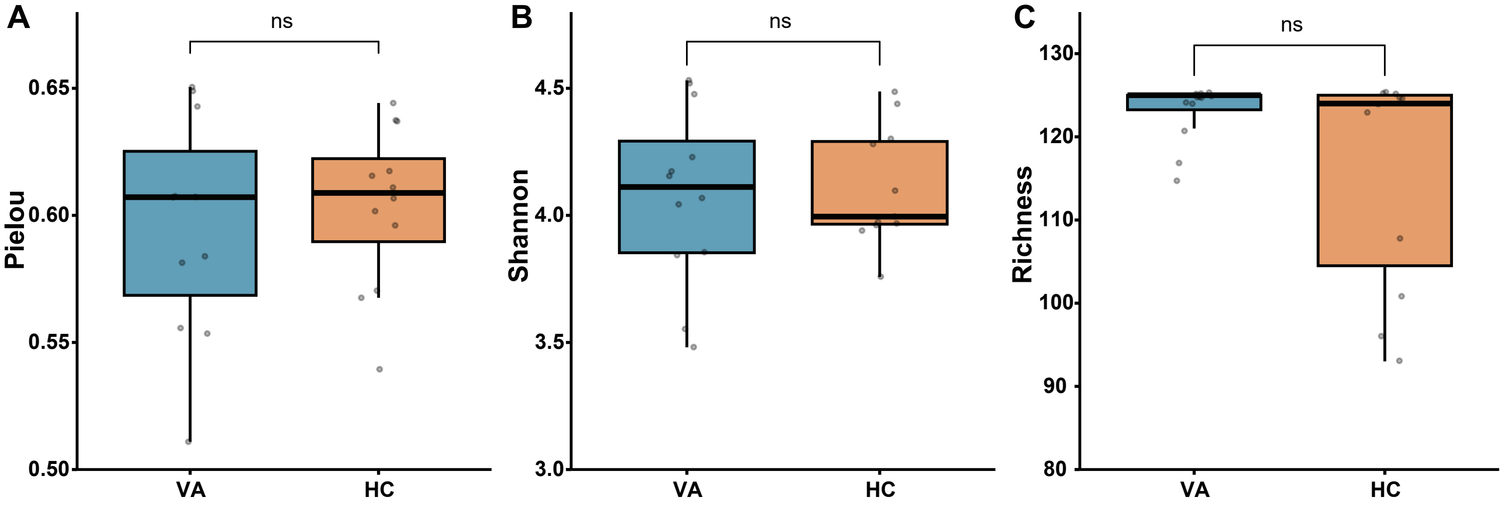

Supplementary Figure 3. Alpha diversity indices (Pielou, Shannon, and Richness) of all 63 papillomavirus-derived lineages, comparing the healthy control and vaginitis groups. Statistical significance was determined using the Wilcoxon rank-sum test. Exact *p*-values: Shannon index ($p = 0.057$), Richness ($p = 0.00056$), Pielou index ($p = 0.011$). Statistical significance thresholds: *$p < 0.05$, **$p < 0.01$, ***$p < 0.001$.

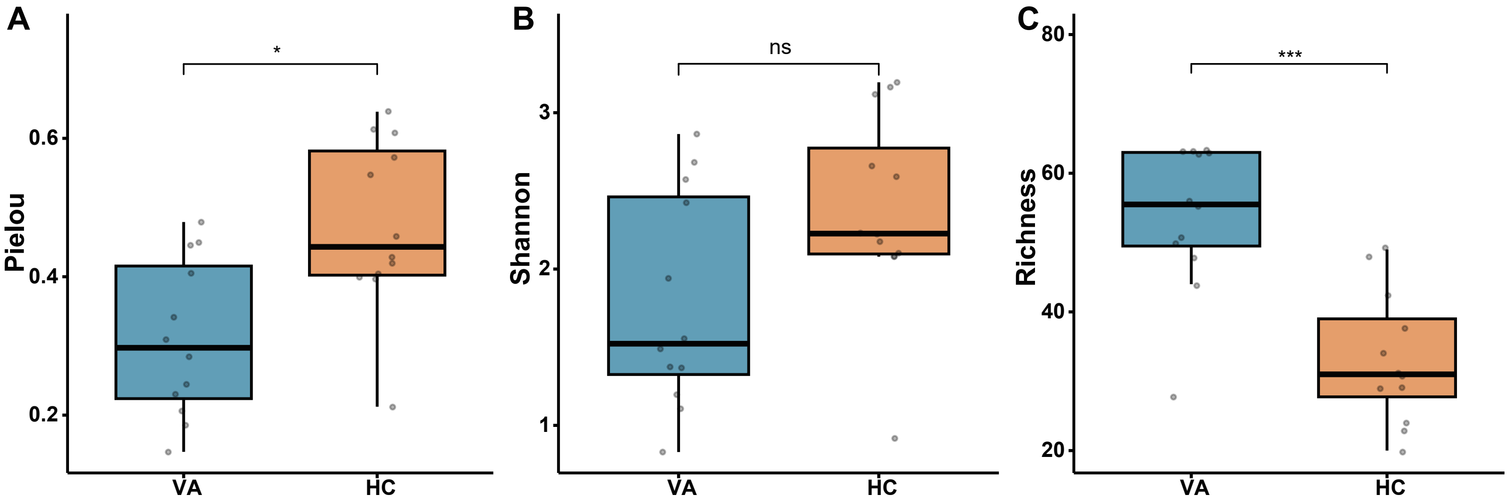

Supplementary Figure 4. Alpha diversity indices (Pielou, Shannon, and Richness) of all 78 phages-derived lineages, comparing the healthy control and vaginitis groups. Statistical significance was determined using the Wilcoxon rank-sum test. Exact *p*-values: Shannon index ($p = 0.2$), Richness ($p = 0.55$), Pielou index ($p = 0.2$). Statistical significance thresholds: *$p < 0.05$, **$p < 0.01$, ***$p < 0.001$.

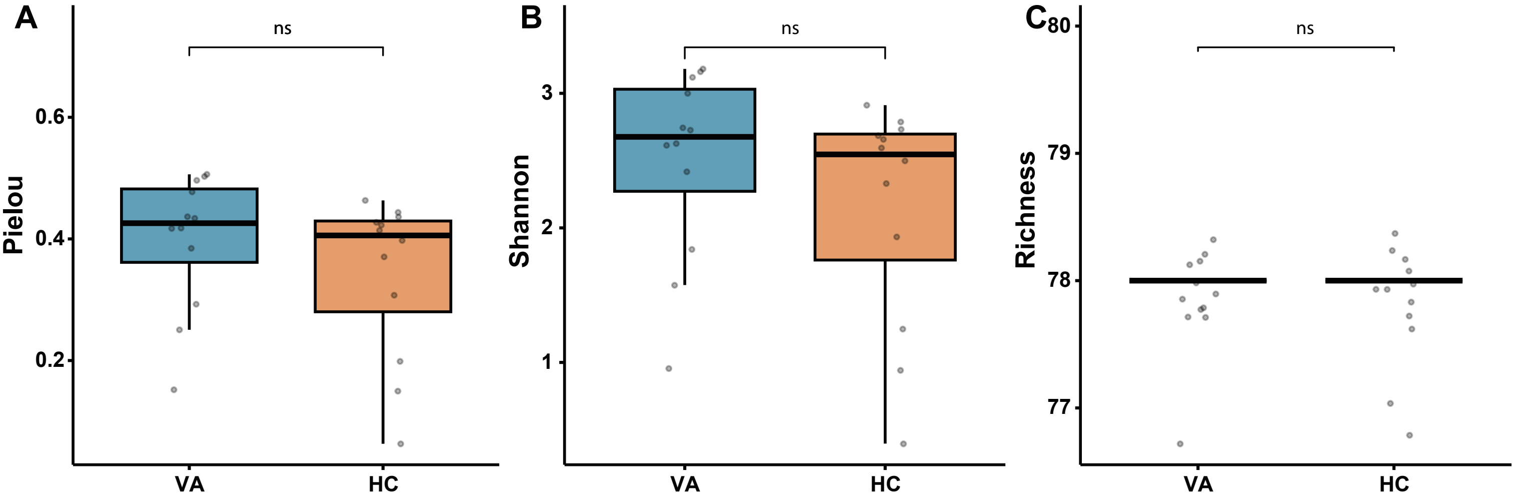

Supplement: Supplementary file 1 [file DataSheet1.pdf]
